# Advantages of Protein A Membrane Chromatography: High Productivity, Strong Impurity Removal Capability, and More

**DOI:** 10.3390/membranes16070222

**Published:** 2026-06-27

**Authors:** Yifeng Li, Hang Zhou, Sherry Gu

**Affiliations:** 1 Downstream Process Development (DSPD), WuXi Biologics, 31 Yiwei Road, Waigaoqiao Free Trade Zone, Shanghai 200131, China; 2 Bioprocess Research and Development (BRD), WuXi Biologics, 31 Yiwei Road, Waigaoqiao Free Trade Zone, Shanghai 200131, China; 3 Global Biologics Development and Operations (GBDO), WuXi Biologics, 31 Yiwei Road, Waigaoqiao Free Trade Zone, Shanghai 200131, China

**Keywords:** aggregate, flexibility, host cell protein (HCP), productivity, Protein A membrane, robustness

## Abstract

Protein A affinity chromatography is the gold standard for antibody purification. In practice, it is mainly performed using resin-based packed bed columns. However, for porous resins, mass transport depends on slow diffusion and, consequently, low flow rates/long residence times are required to achieve decent binding capacities (i.e., >30 mg/mL), which leads to long processing time and low productivity. Recently, Protein A membranes have emerged as a promising alternative to Protein A columns. Membrane adsorbers, which have large pores, enable convection-based mass transport and allow high dynamic binding capacity to be achieved at a relatively short residence time (on the order of seconds). Hence, Protein A membranes can be operated at high flow rates and gain significantly improved productivity. In addition to high productivity, we recently found that Protein A membranes possess a much stronger impurity-removing capability than Protein A columns. This review introduces the advantages that Protein A membrane chromatography offers, especially those that have not been well recognized before. These advantages allow high-quality products to be obtained with significantly reduced costs.

## 1. Introduction

Protein A column chromatography has been widely used as the initial capture step for antibody and Fc-fusion protein purification [1,2,3]. However, for Protein A resins in a packed-bed column, slow intraparticle diffusion limits the rate of mass transport [4]. In addition, the pressure drop across the packed bed is usually high. These two characteristics necessitate low flow rates, resulting in long processing times and low productivity. Different from resins, membrane adsorbers have large pores, and solute transport is primarily driven by quick convection [5,6,7,8,9,10]. Generally, membrane chromatography can be conducted under high flow rates with low pressures. Thus, membrane-based adsorbents can overcome the diffusion limitation and high-pressure issue of resin-based columns.

The first Protein A membrane, in which Protein A ligands were immobilized on membrane supports, appeared more than thirty years ago [11]. However, the early versions had much lower binding capacity compared to resins, which limits their industrial application [11,12,13,14,15,16]. The binding capacity of the Protein A membrane was significantly improved when new support materials were used for ligand immobilization [17]. In recent years, with the continued improvement in binding capacity, Protein A membranes started to become a feasible alternative to resin-based Protein A columns. In comparison to the Protein A column, the Protein A membrane provides several obvious advantages, including high productivity, low pressure drops, small facility footprint, and disposability [18,19,20,21].

While Protein A membranes offer multiple advantages over conventional columns, they currently have a couple of drawbacks. Firstly, Protein A membranes have a lower dynamic binding capacity than Protein A resins (~30 mg/mL vs. >50 mg/mL). Nevertheless, the membrane’s short cycle time/high productivity can partially compensate for this issue. Secondly, Protein A membranes have an increased elution volume (approximately five- to sixfold larger than that of column eluate), which is generally assumed to be caused by the membrane’s large dead volume to stationary phase ratio [20,21]. The increased elution volume leads to undesired sample dilution, increased buffer consumption, and handling challenges at a large scale. It is expected that this issue will be ultimately solved/mitigated by improving the design of the membrane chamber. A temporary solution is to incorporate an ultrafiltration step to concentrate the Protein A membrane eluate [22].

Despite the above-mentioned shortcomings, Protein A membranes have shown great potential as a more productive and efficient alternative to Protein A resins. It is worth mentioning that we recently demonstrated that Protein A membranes exhibit a much stronger impurity-removing capability than Protein A columns [23,24,25,26,27], a remarkable advantage that had not been recognized before. In the following sections, the distinct advantages of Protein A membranes over Protein A columns will be introduced.

## 2. High Productivity and Low Cost

For Protein A membranes, dynamic binding capacity is less dependent on residence time, as proteins can quickly reach their binding sites through convective transport [18,19,20,21]. Therefore, Protein A membranes can be operated at high flow rates and complete a cycle in less than 20 min. In comparison to resin-based columns, whose typical cycle time is 5–6 h, the Protein A membrane increases productivity by more than tenfold. Using the Protein A membrane, a significantly increased number of cycles (e.g., >60) can be conducted within the same time frame required for conducting 3–4 column runs. This significantly reduces the volume of media required for processing the same amount of feed material. For example, while 32.0 L of Protein A resin is required to process a 2000 L batch of clarified harvest at manufacturing scale, a 3.2 L or even a 0.8 L Protein A membrane is sufficient to process the same batch, resulting in up to fortyfold less Protein A media consumption [21].

In common practice, large Protein A columns are used to meet the process time requirement because of their low productivity. Given the high cost of Protein A resins, cost efficiency is only achieved when the resin media’s lifetime is fully utilized. However, the full utilization of a Protein A resin’s lifetime is not always realizable due to low product demand and failure in clinical development. The Protein A membrane can be used for up to 200 cycles before disposal [19,21]. When a right-sized membrane is selected, it is possible to have its lifetime be fully utilized in one or two batches of purification [21]. Thus, replacing the Protein A column with the Protein A membrane can greatly reduce capital expenditures. In addition, the Protein A membrane is a ready-to-use and disposable device, and, therefore, its use eliminates resin-related column packing, unpacking, storage, cleaning, and cleaning validation, which further reduces operational expenses.

## 3. Strong Impurity Removing Capability

In general, resin-based Protein A columns are good at removing process-related impurities, such as DNA and host cell proteins (HCPs), but poor at removing product-related impurities (e.g., aggregates). Recently, we found that the Protein A membrane provides much better resolution than the Protein A column under similar conditions [23,24,25,26]. With the improved resolution, the Protein A membrane can effectively remove fragments due to truncation or incomplete assembly (e.g., single-arm antibody and half-antibody) and aggregates. In addition, by conducting the wash step introduced to disrupt HCP–antibody interactions and the elution step at low and high flow rates, respectively, the Protein A membrane can reduce HCPs to a much lower level than the Protein A column [27].

### 3.1. Fragment Separation

Recombinant antibody expressed in CHO cells can undergo protease cleavage around the hinge region, resulting in a single-arm byproduct (antibody losing one of its Fab arms). This byproduct contains the same Fc region as the intact antibody but different numbers of heavy chain variable domains (VHs). For most antibodies, their VHs belong to the VH3 family. Due to the difference in the number of VH3 domains between the byproduct and the product (one vs. two), MabSelect PrismA, a Protein A resin showing affinity toward both the Fc region and the VH3 domain, provides a certain degree of resolution between the single-arm byproduct and the product [23]. We found that Fibro PrismA, a Protein A membrane from Cytiva that shares the same Protein A ligand with MabSelect PrismA, provided a better resolution than its resin counterpart [23]. Fibro PrismA membrane provided a more complete clearance of the single-arm byproduct while maintaining good recovery of the intact product.

Half-antibody is a byproduct frequently found during the production of asymmetric bispecific antibodies (bsAbs) [28,29]. We previously showed that the Protein A column can separate this byproduct from the intact bsAb under optimized conditions [30]. The difference in the number of Protein A binding sites between the byproduct and the product forms the basis for separation. The half-antibody contains half of the Fc domain (hence one Protein A-binding site), whereas the intact bsAb contains the full Fc region (hence two Protein A-binding sites). Consequently, the half-antibody binds to Protein A resin more weakly than the intact bsAb. However, under regular linear pH gradient elution, only limited resolution was achieved. The resolution between half-antibody and the intact bsAb was significantly improved by adding 500 mM sodium chloride to the mobile phase [30]. Recently, we found that Sartorius’ Sartobind Rapid A Protein A membrane provided a much better resolution than the Protein A resin [24]. A more complete separation of the half-antibody byproduct was achieved under linear pH gradient elution when the mobile phase contained 200 mM sodium chloride. The good separation achieved under linear pH gradient elution allowed the stepwise elution to be developed with ease. Like in column chromatography, under stepwise elution, the half-antibody byproduct was removed by an intermediate pH wash. The higher resolution provided by the Sartobind Rapid A membrane allowed for a more complete half-antibody removal and a higher step yield [24].

### 3.2. Aggregate Removal

**Figure 1:**
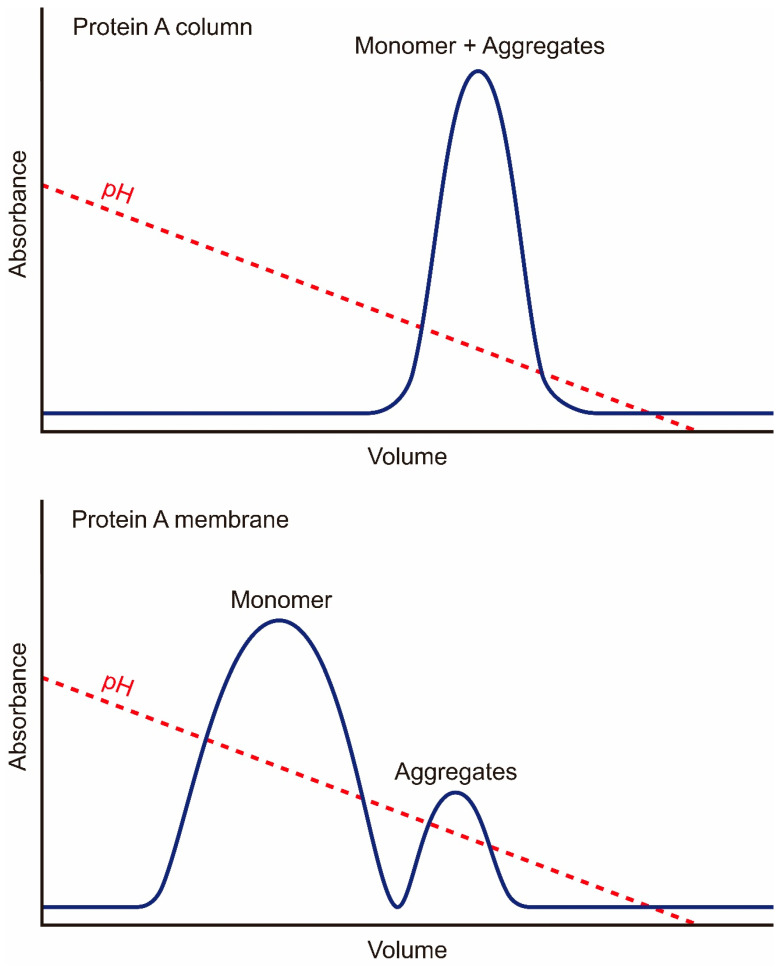
Schematic chromatograms illustrating the difference between the Protein A column and the Protein A membrane in aggregate separation. Top and bottom, Protein A column and Protein A membrane, respectively. For both media, the same loading material and a similar linear pH gradient elution were applied. While the resin-based Protein A column generally lacks the capability to separate co-binding aggregates, the Protein A membrane can provide complete separation, reducing aggregate content from 16.8% (in the load) to 0.1% (in the main elution peak) [25].

We previously showed that the resin-based Protein A column removes large aggregates by excluding them from binding (large aggregates are too big to enter the pores of the resin beads and therefore cannot access the ligands inside) [31,32]. However, Protein A resins typically lack the capability to separate monomers from co-binding small aggregates [33,34]. Consequently, small aggregate removal almost exclusively relies on post-capture polishing steps [35,36,37,38,39,40,41]. For cases where small aggregate contents are high, entirely relying on the polishing steps for removal leaves the downstream processes with poor robustness. Recently, we made an exciting finding that the Protein A membrane possesses a greatly improved aggregate separation capability [25]. For an aggregation-prone mAb, its culture harvest contained approximately 17% of aggregates. When this culture harvest was processed by a Protein A column under linear pH gradient elution, the profile contained a single elution peak, and the aggregate content in the elution pool was approximately 11% (Figure 1, top). When the same culture harvest was processed by the Sartobind Rapid A membrane, the chromatogram contained two well-separated peaks, and the aggregate content in the main elution peak was reduced to 0.1% (aggregates were mainly found in the secondary peak) (Figure 1, bottom) [25]. For the Protein A membrane chromatography, a stepwise elution protocol was subsequently developed, and under these conditions, the aggregate content in the elution pool was 0.2% [25]. Thus, the Sartobind Rapid A membrane removed nearly all aggregates in the load under both linear and stepwise gradient elution, which is a remarkable improvement in comparison to the Protein A column (99% removal vs. 35% removal).

We confirmed Sartobind Rapid A membrane’s strong aggregate separation capability with four additional cases. This Protein A membrane consistently reduced aggregates from greater than 20% to 1–2% [25]. To test the robustness of Sartobind Rapid A-mediated aggregate separation, we also challenged this Protein A membrane with four artificial samples whose aggregate contents ranged from 15 to 60% [26]. The Sartobind Rapid A membrane effectively removed most aggregates in all samples, regardless of the percentage of aggregates in them. These data indicated that aggregate separation by Sartobind Rapid A membrane is not only effective but also highly robust [26].

The strong aggregate separation capability is not limited to the Sartobind Rapid A membrane. Two other Protein A membranes, Fibro PrismA from Cytiva and Pultrix ProA from Cobetter, have shown similar potential [26]. Thus, improved aggregate separation capability is a general property of the Protein A membrane. Although the major difference between the Protein A column and the Protein A membrane lies in their operating flow rates (the residence times for the former and the latter are measured in minutes and seconds, respectively), we have ruled out high flow rate/short residence time as the reason for Protein A membrane’s improved aggregate separation capability by showing that the Sartobind Rapid A membrane exhibited identical elution profiles under different residence times (i.e., 12, 60 and 120 s) [25]. Instead, we found that including certain amounts of salt (e.g., >50 mM sodium chloride) in the mobile phase is critical for the Sartobind Rapid A membrane to achieve good monomer–aggregate separation [25,26]. Among the three commonly used mobile phase additives (i.e., sodium chloride, calcium chloride, and arginine hydrochloride) for improving Protein A chromatography resolution, 150 mM sodium chloride has been shown to provide the best aggregate separation [26]. Interestingly, while sodium chloride enhances antibody binding to the Protein A column [34], it weakens antibody binding to the Protein A membrane [26]. Furthermore, sodium chloride reduces the retention time of monomers to a larger degree than that of aggregates, thereby leading to an improved monomer–aggregate separation [26]. Currently, Protein A membrane’s superior resolution and aggregate separation capability are not fully understood. In fact, before we discovered this unique property, none of the Protein A membrane manufacturers/vendors ever realized it (this is not surprising, as manufacturers typically test their products with limited mAb samples, whose byproduct profiles are usually not complex). As Protein A membranes and Protein A resins share similar Protein A ligands, we speculate that Protein A membranes’ greatly improved resolution is likely attributed to membrane material and/or membrane design. In a recent article, the authors compared a Protein A membrane and a Protein A resin, both of which have the same high pH eluting Protein A ligand immobilized to their supporting matrix, on aggregate separation [42]. As an equally good monomer–aggregate separation was achieved by both the membrane and the resin, the authors concluded that the ligand rather than membrane architecture is the main reason for the improved aggregate separation capability of the Protein A membrane [42]. However, as the Protein A resin based on a high pH eluting ligand shows enhanced aggregate separation in comparison to its regular counterparts [43,44], the impact of membrane material/design on monomer–aggregate separation could be concealed. A comparison between membrane and resin based on the same regular low pH eluting Protein A ligand, which generally lacks aggregate separation capability, would be more appropriate and informative. Overall, further investigation is needed to elucidate the mechanism through which the Protein A membrane gains its improved resolution.

### 3.3. HCP Clearance

In the purification of recombinant antibodies and Fc-fusion proteins, HCPs represent a major class of process-related impurities. As HCPs can cause both safety and efficacy issues, they need to be reduced to an acceptable level (i.e., <100 ppm) by the downstream process [45,46,47,48,49]. Protein A affinity chromatography, owing to its high specificity toward antibodies, can remove more than 90% of HCPs in the feed material, which makes this step the most effective one for HCP clearance among all unit operations in a typical downstream process [1]. Given the fact that the ligands in all Protein A resins are derived from native Protein A, it is not surprising that Protein A resins from different vendors generally provide comparable HCP clearance [50].

The Protein A membrane, as an alternative to the Protein A resin, is expected to show similar HCP-removing capabilities. However, limited and conflicting information was found from different resources in this regard [51,52]. In one study, Gehrmann et al. compared three Protein A membranes (Sartobind Rapid A from Sartorius (Göttingen, Germany), HiTrap Fibro PrismA from Cytiva (Marlborough, MA, USA), and GORE Protein Capture Device from GORE (Newark, DE, USA)) with one Protein A resin (MabSelect PrismA from Cytiva) and showed that all membranes provided better HCP clearance than the resin [51]. In another study, Pasquier et al. compared four Protein A membranes (the same three as those used in the first study and a prototype from Merck (Rahway, NJ, USA)) with two Protein A resins (MabSelect SuRe LX from Cytiva and Amsphere A3 from JSR Life Sciences (Sunnyvale, CA, USA)) and reached an opposite conclusion: both resins outperformed membranes for HCP clearance [52]. For both reports, the observation was made on a limited number of case studies (one and two case studies for the first and the second reports, respectively).

Recently, we compared the Sartobind Rapid A membrane with the MabSelect SuRe LX resin on HCP clearance. To obtain more convincing and accurate results, we conducted the comparison study using culture harvests from ten projects whose target molecules included four mAbs, four bsAbs, a trispecific antibody (TsAb), and a Fc-fusion protein. In all cases but one, the HCP levels of membrane eluates are lower than or comparable to those of column eluates [27]. For that exceptional case, the MabSelect SuRe LX column provided better HCP clearance than the Sartobind Rapid A membrane. An explanation for the membranes’ better performance in general and the exceptional case is highly anticipated. For that exceptional case, enhancing the wash step by applying a high pH or adding sodium caprylate to the wash buffer had no effect on HCP removal by the Protein A membrane, although both optimizations greatly improved HCP clearance by the Protein A column [27]. Nevertheless, in this case, the HCP level in the Sartobind Rapid A membrane eluate was significantly reduced when the flow rate of the high salt wash step was lowered to reach a corresponding residence time of five minutes. This suggests that flow rate is a critical factor for HCP clearance in Protein A membrane chromatography [27].

According to previous studies, there are two pathways through which HCPs get copurified with the target antibody during Protein A chromatography [53,54]. Firstly, heteroaggregates formed with DNA-histone core, non-histone HCPs, and misfolded mAbs bind strongly to Protein A resin, and a subset of the associated HCPs is leached during the low pH elution, which accounts for a major portion of HCPs found in the Protein A eluate [55,56,57]. HCP leaching during elution can be minimized by applying mild conditions. In consistency with this, the eluate of Jetted A50 HipH, a Protein A resin that supports elution at pH 4.7, contains a lower level of HCPs compared to that of regular Protein A resins, which typically require a lower pH (i.e., ~3.6) for elution [58,59]. Secondly, some HCPs are associated with the target antibody through different forces (e.g., hydrogen bonding, hydrophobic effects, and electrostatic interactions) [60,61,62,63,64]. For removing this group of HCPs, it is critical to disrupt the HCP–antibody interactions first. In Protein A chromatography, promotion of HCP–antibody disassociation is usually achieved by adopting a high pH wash or adding different reagents (e.g., amino acids, chaotropic salts, organic solvents, and detergents) to the wash buffer [60].

**Figure 2:**
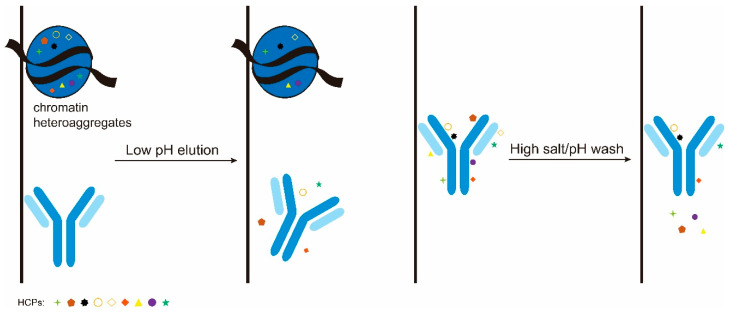
Schematic illustration of the double-edged sword effect of the Protein A membrane’s high flow rate on HCP clearance. The high flow rate adopted by the Protein A membrane chromatography simultaneously serves as a favorable and an unfavorable factor for minimizing HCP copurification. **Left**: elution under high flow rate reduces exposure to low pH and minimizes HCP leaching from chromatin heteroaggregates. **Right**: washing under a high flow rate reduces exposure to high salt/pH, which is introduced to disrupt HCP–antibody interactions, and hinders HCP–antibody disassociation.

We speculated that the high flow rate adopted by Protein A membrane chromatography simultaneously serves as a favorable and an unfavorable factor for minimizing HCP copurification [27]. On one hand, the short residence time reduces HCP leaching during elution (Figure 2, left); on the other hand, the short residence time hinders HCP–antibody disassociation during washing (the contact time is insufficient for the wash condition to exert its effect on disrupting HCP–antibody interactions) (Figure 2, right). This explains the inconsistent results of previous studies and why optimizing the wash condition had no effect on the Protein A membrane. In cases where the interactions between HCPs and the target antibody are relatively weak (this is likely true for most cases), the advantage of fast elution overwhelms the disadvantage of fast wash, resulting in better HCP clearance than resin/column. In cases where strong interactions exist between HCPs and the target antibody, the disadvantage of fast wash has a predominant effect, resulting in inferior HCP clearance compared to resin/column. Thus, for Protein A membrane chromatography to achieve minimum HCP copurification, the wash step aimed at disrupting HCP–antibody interactions should be conducted at a lower flow rate while all other steps are conducted at membrane chromatography’s typical high flow rate [27].

## 4. Improved Flexibility and Simplicity

In comparison to the Protein A column, the Protein A membrane has a much shorter cycle time (>5 h vs. <20 min). Protein A membrane’s high productivity offers great flexibility to the process in addition to saving costs (as shown in the previous sections). For example, its single-use nature allows rapid changeovers and facilitates multi-product manufacturing. In addition, the use of the Protein A membrane can improve process robustness and allow continuous capture to be realized in a more convenient way.

### 4.1. Enhanced Robustness

Protein A membrane offers great flexibility, which can significantly improve the robustness of a process. In the case where half-antibody removal is achieved by a pre-elution wash during Protein A column chromatography, the performance of this key wash step is highly sensitive to the loading density [30]. Typically, the wash condition is developed under an average loading density (e.g., 30 mg/mL), which aims to maximize half-antibody removal while maintaining good product yield. However, when the loading density deviates from that under which the best wash condition is reached, which is unavoidable in practice, the developed wash condition leads to either inadequate byproduct removal (at a lower loading density) or product loss (at a higher loading density) [30]. Thus, the process, which relies on a defined wash for half-antibody removal, lacks sufficient robustness to variations in harvest titer (as the column size and cycle number are usually predetermined, variation in harvest titer requires adjustment of each cycle’s loading density).

The Protein A membrane provides a solution to the above low robustness issue [24]. Its short cycle time allows variation in harvest titer to be dealt with by adjusting the cycle number instead of loading density. For all cycles but the last one, their loading densities can be fixed to that under which the optimal wash condition is developed. For the last cycle, although its loading can deviate from the desired amount, resulting in compromised quality or yield, its impact on the overall quality and yield is negligible as its eluate only accounts for a small portion. This holds true not only for the Protein A membrane but also for other types of bind-elute mode membrane chromatography (e.g., cation exchange membrane) [65]. Conducting nearly all cycles under a defined loading amount avoids loading density fluctuation-induced performance change and greatly minimizes run-to-run variation.

### 4.2. Simple Configuration for Continuous Capture

In comparison to traditional batch-mode processing, multi-column continuous Protein A chromatography can significantly improve productivity and reduce resin costs [66,67,68]. For a typical periodic counter-current chromatography (PCC), three or more columns are required. A key feature is that two columns are connected in series during loading, so that breakthrough from the first column is captured by the second column. This allows the first column to be loaded close to the resin’s static binding capacity, resulting in more efficient resin utilization. Nevertheless, such a design increases process complexity and the chance of contamination. While the Protein A membrane can support continuous capture in a similar way as the column, its short cycle time allows the same purpose to be served with a simpler configuration [17,69,70,71]. Specifically, continuous capture can be realized with just two Protein A membranes, which are operated in parallel [69,70]. While one Protein A membrane is loaded, the other Protein A membrane is washed, eluted, regenerated, and equilibrated. Thus, the Protein A membrane provides a more convenient way to support continuous capture. In fact, with a surge tank, we even used a single Protein A membrane to support the continuous capture of a 50 L scale perfusion culture for nine days in a pilot plant. A comparison between this and a hypothetical three-column approach is provided in Table 1.

## 5. Closing Remarks

In recent years, the advantages and potential of convective membrane chromatography have gained increasing appreciation. For the Protein A membrane, its most notable advantage is the significantly increased productivity. Recently, we demonstrated that Protein A membranes also show much stronger impurity-removing capabilities than Protein A columns. While high productivity allows a large amount of culture harvest to be processed using a relatively small-sized membrane, a strong impurity removing capability potentially allows the number of polishing steps to be reduced from two to one, both leading to great savings in cost. In addition, the flexibility and simplicity that the Protein A membrane offers can improve process robustness and minimize run-to-run variation.

The Protein A membrane’s high productivity and several other initially recognized advantages (e.g., small facility footprint and disposability) have attracted increasing attention. Multiple vendors (e.g., Cobetter, Cytiva, GORE, Merck, Purilogics, Sartorius, and Thermo Fisher) are actively developing their own Protein A membrane products. Sartorius promises that its second-generation Sartobind Rapid A will have higher binding capacity and smaller elution volume. Cobetter, a filter supplier, recently launched its brand Pultrix ProA at both small and large scales. Protein A membrane’s strong impurity-removing capability was only recently discovered by us and has not been widely known to relevant researchers/users yet. We expect that this newly discovered advantage will attract more developers and end users. In addition, like in resins, ligands that recognize domains other than Fc can be used to generate affinity membranes with distinct specificities. While it is unlikely that Protein A membranes will replace Protein A resins to a large degree soon, with the improvement in binding capacity, elution volume, scalability, and the increase in diversity, Protein A membranes and affinity membranes in general will certainly become more competitive and have a broader application in the biopharmaceutical industry.

## Figures and Tables

**Table 1:** A comparison between the actual single-membrane mode and the hypothetical three-column mode continuous capture.

Items	Single-Membrane Mode	Three-Column Mode
Media Volume (L)	0.2	~1.6 a
Load Density (g/L)	36	50
Residence Time (min)	0.2 b	2 c
Cycle Time (min)	12.7	57.3
Cycle No. (per day)	36	10
Total Cycle No.	324	90
Processing Time (hours per day)	7.6	9.6
Total Processing Time (hours)	68.4	86.4
Total Buffer Consumption (L)	6565.7	3216.8
Elution Volume (MV/CV per cycle)	8	2
Elution Volume (L per cycle)	1.6	1.0
Total Elution Volume (L)	518	90
Productivity (g/L/h) d	170.5	17.4

A total volume of three identical columns. The column has a dimension of 14 cm (D) × 3.4 cm (H); b 0.2 min for load and elution, and 0.1 min for all other steps; c 2 min for load and elution, and 1 min for all other steps; d was calculated based on a total protein mass of 2331 g (titer: 5.18 g/L, daily harvest volume: 50 L, duration: 9 days). MV, membrane volume and CV, column volume.

## Data Availability

No new data were created or analyzed in this study. Data sharing is not applicable to this article.

## Abbreviations

HCP	host cell protein
VH	heavy chain variable domain
bsAb	bispecific antibody
TsAb	trispecific antibody
PCC	periodic counter-current chromatography
